# Hepatocyte growth factor reduces sensitivity to the epidermal growth factor receptor-tyrosine kinase inhibitor, gefitinib, in lung adenocarcinoma cells harboring wild-type *EGFR*

**DOI:** 10.18632/oncotarget.7586

**Published:** 2016-02-22

**Authors:** Hua Yang, Rong Wang, Shunli Peng, Longhua Chen, Qi Li, Wei Wang

**Affiliations:** ^1^ Department of Radiation Oncology, Nanfang Hospital, Southern Medical University, Guangzhou, People's Republic of China; ^2^ Oncology Department, Nanhai Hospital, Southern Medical University, Fuoshan, People's Republic of China; ^3^ Guangdong Provincial Key Laboratory of Gastroenterology, Department of Gastroenterology, Nanfang Hospital, Southern Medical University, Guangzhou, People's Republic of China

**Keywords:** EGFR-TKI, drug resistance, HGF, lung cancer, wild-type EGFR

## Abstract

Epidermal growth factor receptor tyrosine kinase inhibitor (EGFR-TKI) therapy is an option for lung cancers harboring wild-type *EGFR* when chemotherapeutic reagents have failed. In this study, we found that the EGFR-TKI, gefitinib, modestly suppressed proliferation of the lung cancer cell lines, A549 and H358, which both harbor wild-type *EGFR*. Treatment with hepatocyte growth factor (HGF) reduced the sensitivity to gefitinib, whereas sensitivity was restored by treatment with an HGF antibody, a MET inhibitor, or depletion of *MET* but not ErbB3 gene. Moreover, both PI3K/mTOR inhibitors and MEK inhibitors suppressed proliferation of A549 cells, whereas only PI3K/mTOR inhibitors effectively suppressed cell viability of *EGFR* mutant PC-9 cells. Our findings suggest that HGF reduced the gefitinib sensitivity through MET and downstream PI3K and MAPK pathways. Combined use of EGFR-TKI and MET inhibitors or inhibition of downstream signaling molecules might be a better second or third line choice for a group of patients with advanced lung cancer harboring wild-type *EGFR*.

## INTRODUCTION

Non-small cell lung cancers (NSCLC) account for about 85% of lung cancers. Among patients with NSCLC, more than 65% present with locally advanced or metastatic disease [1]. Unfortunately, less than 15% of patients with lung cancer survive more than five years. Over the past decade, the identification of specific genetic alterations, such as oncogenic drivers, in lung cancers has lead to a new period of targeted therapy [2]. Targeting oncogenic drivers, such as epidermal growth factor receptor (*EGFR*) and anaplastic lymphoma kinase (*ALK*), has brought the most encouraging improvements in lung cancer treatment. Clinical trials of targeted therapy, such as with EGFR-tyrosine kinase inhibitor (EGFR-TKI) therapy in *EGFR* mutant NSCLC and crizotinib therapy in *ALK* rearranged NSCLC, have demonstrated major improvements in treatment response, quality of life, and progression-free survival compared to chemotherapy [3–5].

EGFR-TKIs, such as erlotinib, gefitinib, and afatinib, are established as initial standard therapies [6–9]. These treatments are particularly effective against NSCLCs harboring activating mutations in *EGFR*, such as the exon 19 deletion and L858R mutation. Activating mutations in *EGFR* are observed in up to 50% of lung adenocarcinomas in Asians and approximately 10% of Caucasians with NSCLC [10]. Although most patients with *EGFR* mutations initially respond to TKI therapy, almost all develop acquired resistance. Therefore, in trinsic and acquired resistance have become serious barriers to the outcomes of patients treated with these reagents.

Many of the EGFR-TKI resistant mechanisms have been revealed. Recent studies using new generation EGFR-TKIs show good efficacy in resistant tumors with the *EGFR* T790M gatekeeper mutation, which accounts for approximately 50% of resistant tumors [11, 12]. Previously, we have reported that hepatocyte growth factor (HGF), the ligand of the MET receptor, induces resistance to gefitinib or new generation EGFR-TKIs in *EGFR* mutant lung adenocarcinomas through the MET/Gab1/PI3K/Akt pathway, without involvement of ErbB3 [13, 14], although ErbB3 was critical in *MET* amplification–induced gefitinib resistance [15]. We also found that the MET inhibitor, E7050, successfully overcame HGF-induced resistance to EGFR-TKIs [16, 17].

For most patients with advanced lung cancer harboring wild-type *EGFR*, chemotherapy is the prior choice [18]. However, resistance to chemotherapeutic reagents usually develops over time and limits the clinical benefit from chemotherapy. Although the effect of EGFR-TKIs in NSCLC patients with wild-type EGFR remains controversial, some clinical data have shown survival benefit derived from EGFR-TKI treatment for patients previously treated with chemotherapy but still developed disease progression [19–22]. Therefore, several guidelines recommend EGFR-TKIs as an option of second-line treatments for NSCLC patients with wild-type *EGFR* [23]. Considering the nearly unavoidable resistance to EGFR-TKIs, we propose that a resistance mechanism may also exist in wild-type *EGFR* lung cancer. If the potential resistance can be identified prior to EGFR-TKI therapy, this specific group of patients may benefit more from EGFR-TKIs.

Since HGF/MET was previously identified as playing a critical role in the resistance mechanism of EGFR-TKIs in *EGFR* mutant NSCLC, we investigated whether HGF also influenced EGFR-TKI sensitivity in lung adenocarcinoma cells harboring wild-type *EGFR*. We found that HGF significantly reduced sensitivity to gefitinib through the PI3K/Akt and MAPK pathways in lung cancer cells with wild-type *EGFR*. A MET inhibitor, PHA-665752, completely restored the sensitivity to EGFR-TKI. The results of this study suggest that combined use of EGFR-TKI and MET inhibitors or inhibition of downstream signaling molecules, such as PI3K or MEK, might be a better second or third-line strategy for a group of patients with advanced lung cancer harboring wild-type *EGFR*.

## RESULTS

### HGF reduces sensitivity to gefitinib in lung adenocarcinoma cells harboring wild-type *EGFR*

**Figure 1:**
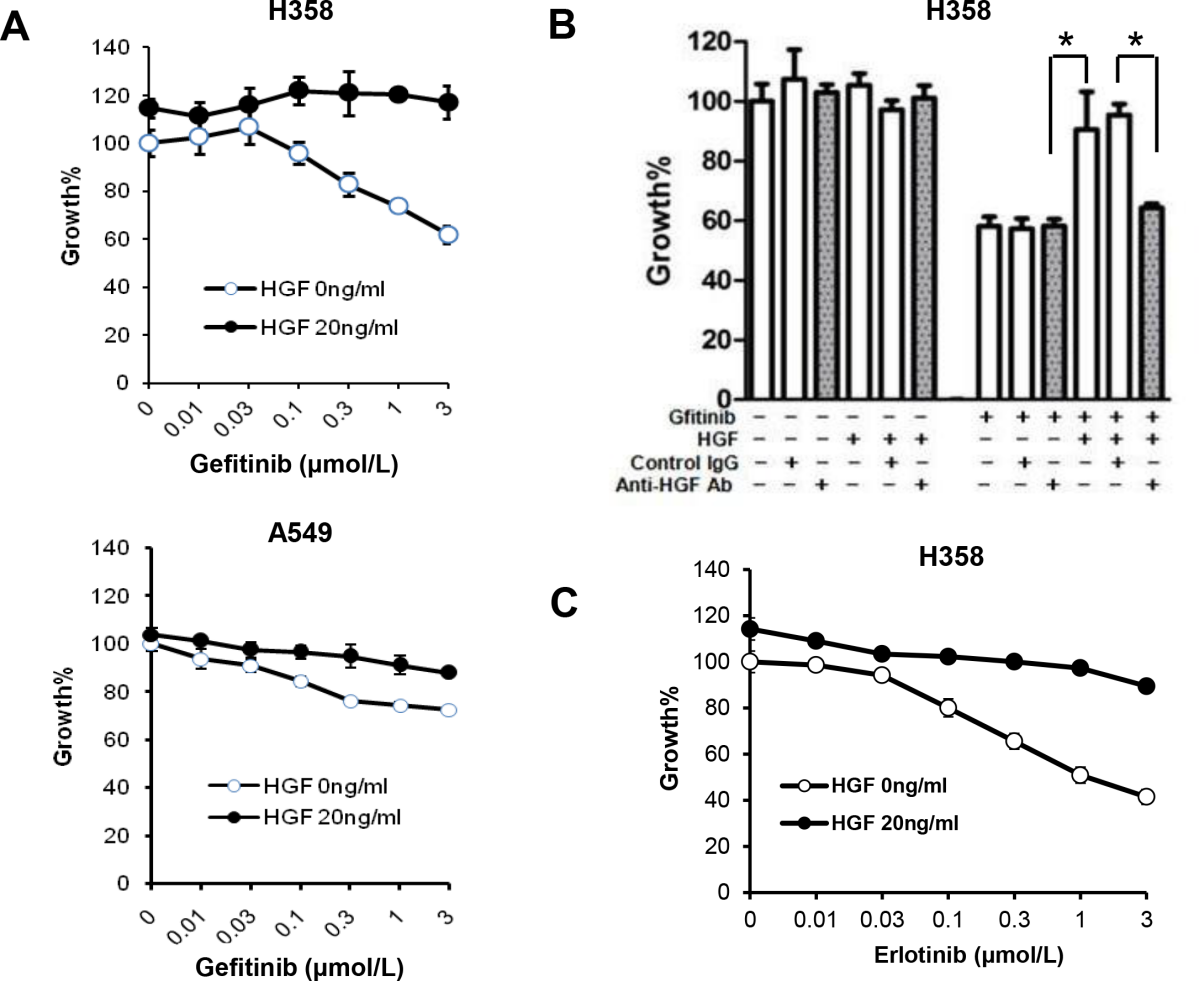
HGF reduces sensitivity to gefitinib in lung adenocarcinoma cells harboring wild-type EGFR (**A**) Treatment with HGF reduces sensitivity to gefitinib in wild-type *EGFR* harboring A549 and H358 cells. Tumor cells were incubated with increasing concentrations of gefitinib and/or HGF, and cell growth was determined after 72 h of treatment by MTT assay. (**B**) Pretreatment of HGF with anti-HGF antibody abrogated the HGF-induced resistance of H358 cells to gefitinib. HGF (20 ng/mL) was pretreated with control IgG (2 μg/mL) or anti-HGF antibody (2 μg/mL) for 1 h. The resultant solutions were added to the cultures of tumor cells with or without gefitinib (1 μmol/L). Cell growth was determined in the same way as in panel A. *P < 0.01. (**C**) HGF reduces sensitivity to erlotinib in H358 cells with wild-type EGFR. Tumor cells were incubated with increasing concentration of gefitinib and/or HGF, and cell growth was determined by MTT assay.

Gefitinib sensitivity was examined in the lung cancer cell lines, H358 and A549, which express wild-type *EGFR*. Both cell lines express a mutant *KRAS* gene that is known as a marker of low sensitivity to EGFR inhibition and chemotherapy [24]. As shown in Figure 1A, cell viability of both H358 and A549 cells were modestly inhibited by gefitinib. Treatment with HGF reduced the sensitivity of both cell lines to gefitinib. The effect of HGF was abrogated by pretreatment with an anti-HGF neutralizing antibody but not control IgG (Figure 1B). In a parallel study, erlotinib suppressed cell viability of H358 cells, but treatment with HGF rescued cells from the effects of erlotinib (Figure 1C). These data indicate that HGF reduced EGFR-TKI sensitivity in lung cancer cells harboring wild-type *EGFR*.

### HGF reduces sensitivity to gefitinib by directly restoring phosphorylation of Akt and ERK1/2

**Figure 2:**
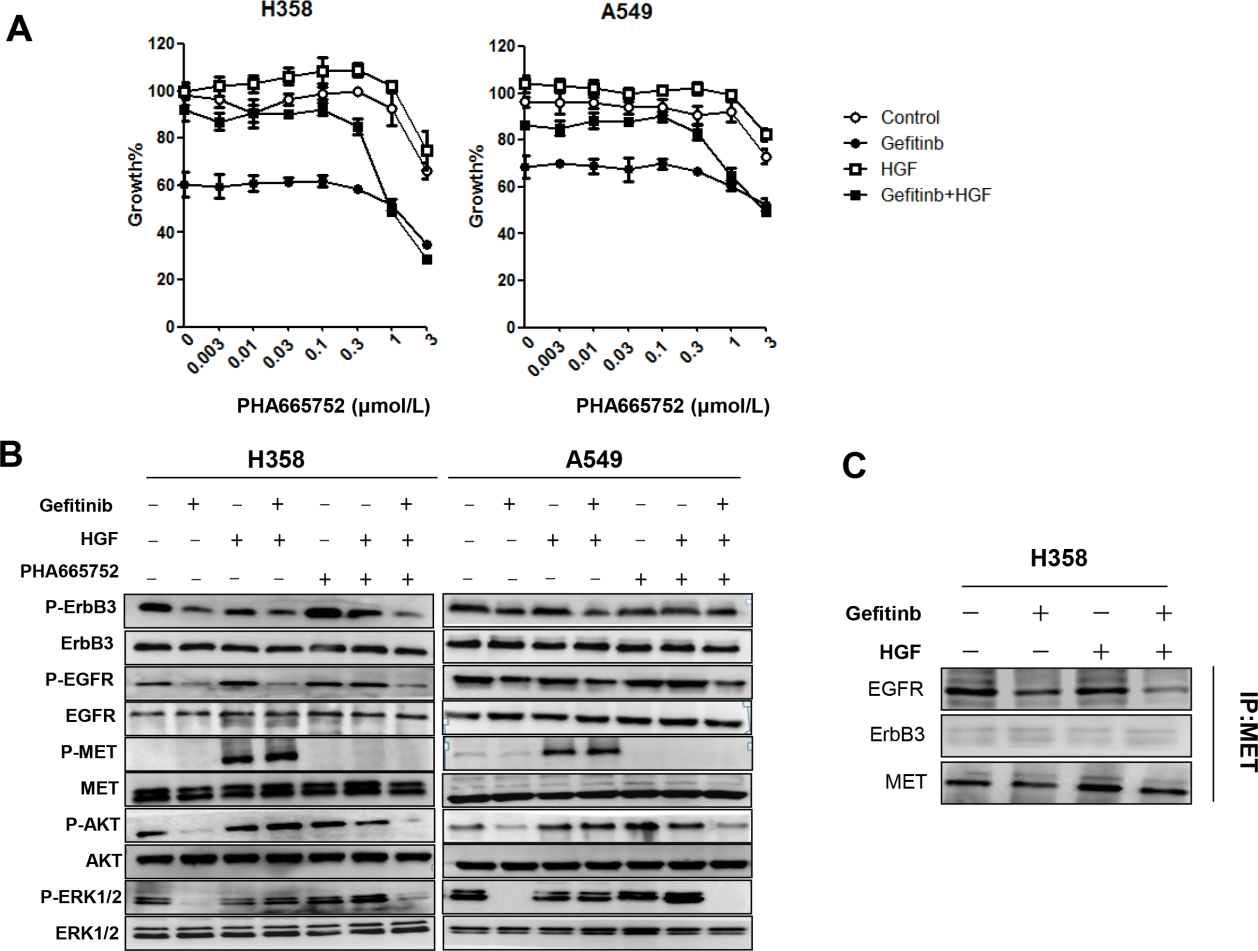
HGF reduces sensitivity to gefitinib by directly restoring the phosphorylation of Akt and ERK1/2 (**A**) H358 and A549 cells were incubated with various concentrations of PHA-665752, with or without HGF (20 ng/mL) and/or gefitinib (1 μmol/L), and cell growth was determined by MTT assay. (**B**) H358 and A549 cells were incubated with HGF (20 ng/mL), PHA-665752 (1 μmol/L), and/or gefitinib (1 μmol/L) for 1 hour. The cell lysates were harvested and phosphorylation of indicated proteins was determined by Western blotting. (**C**) Cell extracts were immunoprecipitated with an antibody to MET. The precipitated proteins were identified by immunoblotting with the indicated antibodies.

Next, we explored whether inhibition of MET, the receptor of HGF, could restore the sensitivity to gefitinib in lung cancer cells with wild-type *EGFR* that were pretreated with HGF. Although the MET inhibitor, PHA-665752, did not affect the growth of H358 or A549 cells at concentrations less than 0.3 μmol/L, it restored the sensitivity of cells to gefitinib in a concentration-dependent manner (Figure 2A).

Although both *MET* gene amplification and HGF treatment has been shown to induce gefitinib resistance in lung cancers with *EGFR* mutations, ErbB3 transactivation is involved only in *MET* amplification but not in HGF-induced resistance [25]. Therefore, to explore the molecular mechanism by which HGF reduces sensitivity to gefitinib in lung cancer harboring wild-type *EGFR*, we examined the phosphorylation status of MET, EGFR, ErbB3, and the downstream signaling pathways by western blotting. H358 and A549 cells expressed EGFR, ErbB3, and MET proteins, and these molecules were constitutively phosphorylated at various levels. The downstream proteins Akt and ERK1/2 were also phosphorylated. Consistent with the cell viability data, gefitinib successfully inhibited the phosphorylation of EGFR, ErbB3, Akt, and ERK1/2 (Figure 2B). Neither HGF nor PHA-665752 treatment alone affected phosphorylation of EGFR or ErbB3. However, HGF stimulated phosphorylation of MET; MET phosphorylation was inhibited by PHA-665752. Importantly, even in the presence of gefitinib, HGF restored phosphorylation of Akt and ERK1/2, but not EGFR or ErbB3 (Figure 2B). Moreover, the combination of PHA-665752 with gefitinib inhibited MET phosphorylation along with Akt and ERK1/2 phosphorylation. These data indicate that HGF reduced sensitivity to gefitinib by restoring MET-mediated phosphorylation of Akt and ERK1/2 in cells harboring wild-type *EGFR*, consistent with previous data examining *EGFR* mutant lung cancer cells.

To investigate the relationship between EGFR, MET and ErbB3, we immunoprecipitated MET and examined its correlation with EGFR and ErbB3 in H358 cells. As shown in Figure 2C, MET was not immunoprecipitated with ErbB3, and neither gefitinib nor HGF induced an association of these two molecules. Conversely, MET was constitutively associated with EGFR, and this association was inhibited by gefitinib, suggesting that the association between EGFR and MET may be correlated by EGFR phosphorylation status. Moreover, HGF treatment failed to restore the association inhibited by gefitinib (Figure 2C). These results indicate that reduced sensitivity to EGFR-TKI with HGF treatment is mediated by the MET signaling pathway but not by ErbB3.

### Specific down-regulation of MET, but not ErbB3, restored sensitivity to gefitinib by inhibiting HGF-induced phosphorylation of Akt and ERK1/2

**Figure 3:**
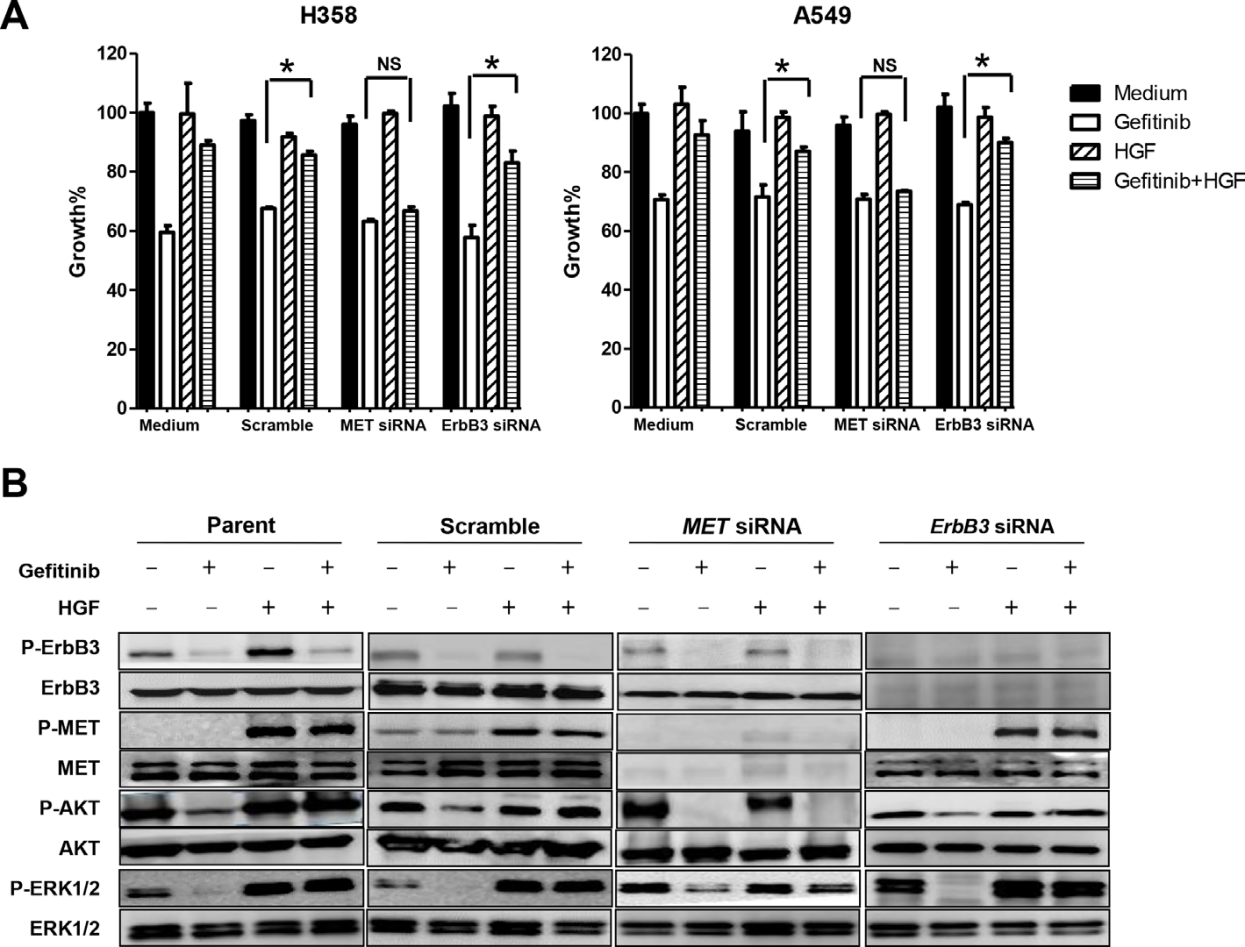
Specific down-regulation of MET, but not ErbB3, restored gefitinib sensitivity by inhibiting HGF induced-phosphorylation of Akt and ERK1/2 (**A**) MET siRNA, but not ErbB3 siRNA, reversed the enhanced gefitinib resistance with HGF. Cell growth in the presence or absence of gefitinib (1 μmol/L) and/or HGF (20 ng/mL) was measured by MTT assay. *P < 0.01. NS: no statistical difference. (**B**) Down-regulation of MET, but not ErbB3, with specific-siRNAs restored HGF-induced phosphorylation of Akt and ERK1/2 in cells treated with gefitinib. Scramble, MET-specific, or ErbB3-specific siRNAs were introduced into H358 cells. After 48 h, the cells were treated with or without gefitinib (1 μmol/L) and/or HGF (20 ng/mL) for 1 h, and then cell extracts were prepared and immunoblotted with the indicated antibodies.

RNAi was used to determine whether MET was responsible for restoring gefitinib sensitivity. Down-regulation of ErbB3 expression by an ErbB3-specific siRNA did not affect either HGF-reduced sensitivity to gefitinib (Figure 3A) or HGF-restored phosphorylation of Akt and ERK1/2 in H358 or A549 cells (Figure 3B). In contrast, down-regulation of MET expression by MET-specific siRNA recovered the sensitivity to gefitinib (Figure 3A), as well as the effect of HGF on the phosphorylation of Akt and ERK1/2 (Figure 3B). These results indicate that HGF reduces sensitivity to gefitinib in lung cancer cells harboring wild-type *EGFR* by restoring the Akt signaling pathway via MET phosphorylation, independently of ErbB3.

### HGF derived from tumor cells reduces sensitivity to gefitinib in lung adenocarcinoma cells harboring wild-type *EGFR*

**Figure 4:**
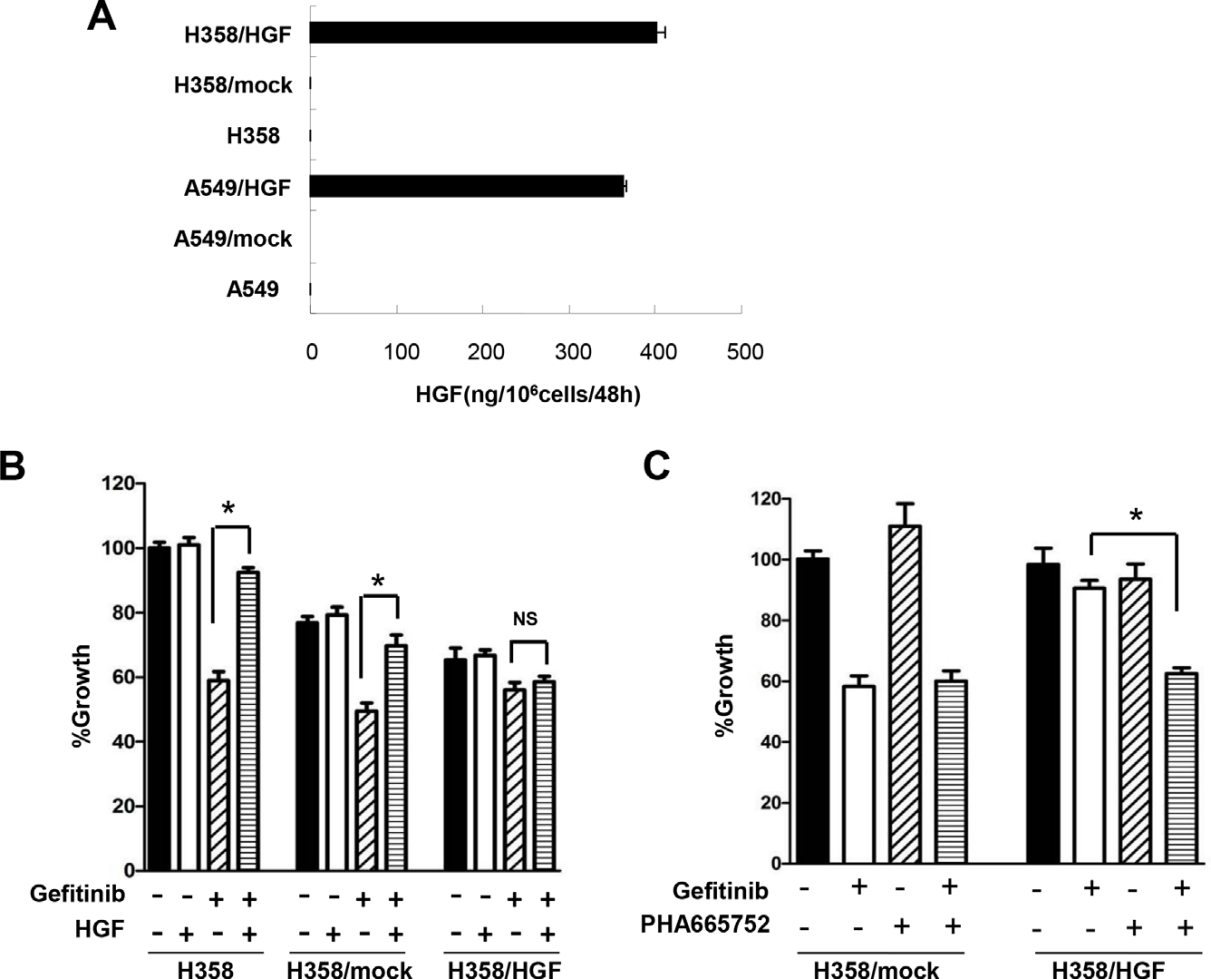
HGF derived from tumor cells reduces sensitivity to gefitinib in lung adenocarcinoma cells harboring wild-type *EGFR* (**A**) HGF production by A549, H358, mock-transfected A549 (A549/mock), mock-transfected H358 (H358/mock), *HGF* gene-transfected A549 (A549/HGF), and *HGF* gene-transfected H358 (H358/HGF) cells. The cells were incubated in medium for 48 h and culture supernatants were harvested. The level of HGF in the supernatants was determined by ELISA. (**B**) *HGF* gene transfection in H358 cells resulted in resistance to gefitinib. H358, H358/mock, and H358/HGF cells were incubated with or without gefitinib (1 μmol/L) in the presence or absence of HGF (20 ng/mL). Cell growth was measured by MTT assay. *P < 0.01. NS: no statistical difference. (**C**) MET inhibition abrogated endogenous HGF-induced gefitinib resistance. H358/mock and H358/HGF cells were incubated with or without gefitinib (1 μmol/L) in the presence or absence of PHA-665752 (1 μmol/L). Cell growth was measured by MTT assay. *P < 0.01.

The *HGF* gene was transfected into lung adenocarcinoma cells. Prior to transfection, H358 and A549 cells secreted undetectable levels of HGF, whereas high levels of HGF were detected in the supernatant of HGF-transfected cells, H358/HGF and A549/HGF (Figure 4A). As expected, H358/HGF cells, but not cells transfected with vector alone (H358/mock), became highly resistant to gefitinib (Figure 4B). Sensitivity to gefitinib was restored by treatment with PHA-665752 (Figure 4C). These results indicate that tumor cell–derived HGF reduces the sensitivity to gefitinib in lung adenocarcinoma cells harboring wild-type *EGFR*.

### Both PI3K/Akt and MAPK signaling pathways are essential to gefitinib resistance in lung cancer cells harboring wild-type *EGFR*

**Figure 5:**
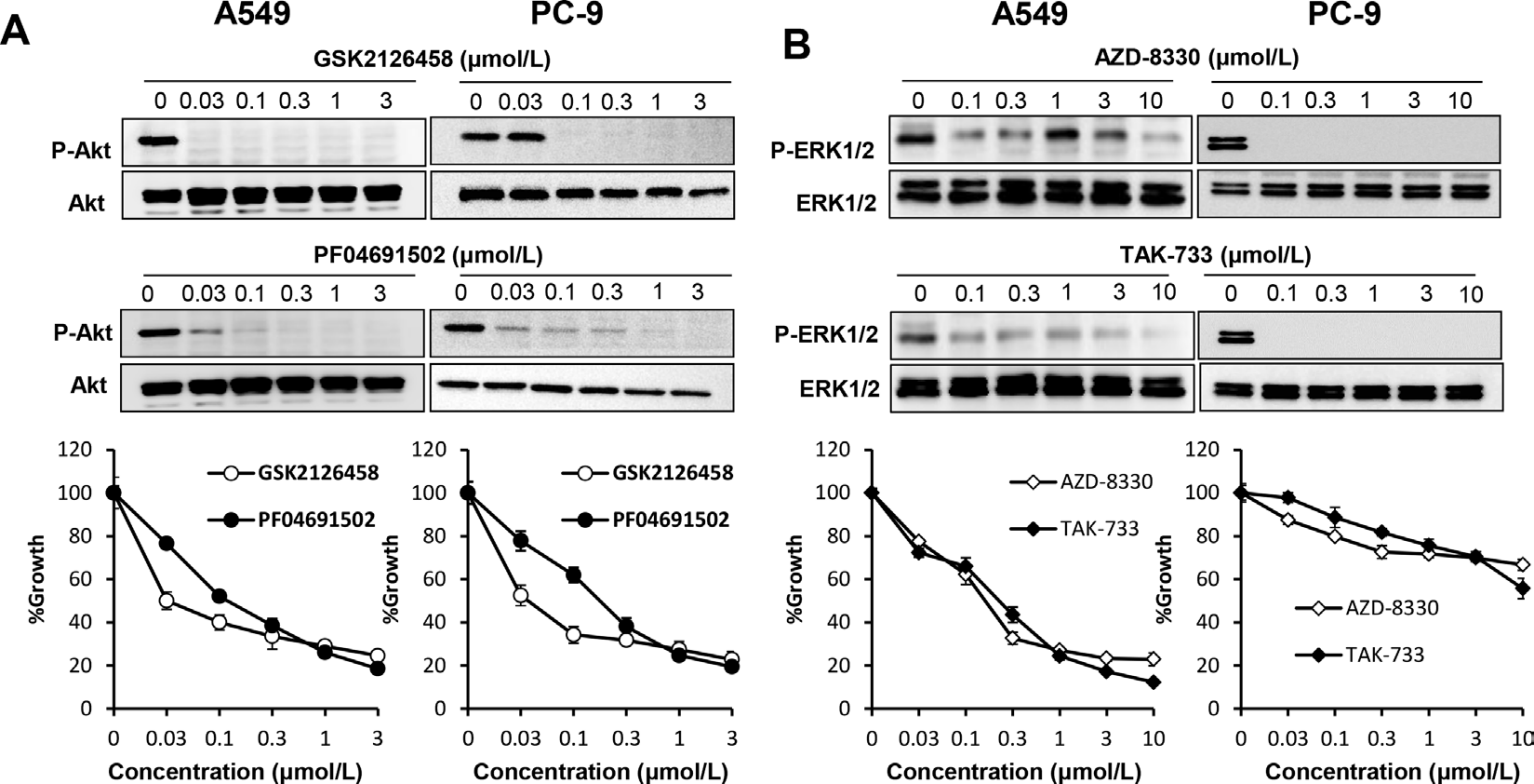
Both PI3K/Akt and MAPK signaling pathways are essential in lung cancer cells harboring wild-type *EGFR* (**A**) PI3K inhibition suppressed proliferation of lung cancer cells harboring mutant or wild-type *EGFR*. A549 and PC-9 cells were incubated in the presence of various concentrations of GSK2126458 or PF04691502. Cell extracts were prepared after 1 h of treatment and immunoblotted with the indicated antibodies (upper panel). Cell growth was measured by MTT assay after 72 h (lower panel). (**B**) MEK inhibition suppressed proliferation of lung cancer cells harboring mutant or wild-type *EGFR*. A549 and PC-9 cells were incubated in the presence of various concentrations of AZD-8330 or TAK-733. Cell extracts were prepared after 1 h of treatment and immunoblotted with the indicated antibodies (upper panel). Cell growth was measured by MTT assay after 72 h (lower panel).

The PI3K/Akt signaling pathway has been reported as a critical component in the acquired resistance to gefitinib in *EGFR* mutant NSCLC cells [26–28]. Therefore, we next sought to determine the importance of the PI3K and MAPK downstream signaling pathways in lung cancer cells with mutant or wild-type *EGFR* using PI3K inhibitors and MEK inhibitors. The PI3K inhibitors, GSK2126458 and PF04691502, strongly inhibited the phosphorylation of Akt. These agents significantly suppressed the proliferation of wild-type *EGFR* A549 cells and *EGFR*-mutant PC-9 cells (Figure 5A). The MEK inhibitors, AZD-8330 and TAK-733, also significantly inhibited the proliferation of A549 cells. However, the inhibitory growth effect of the MEK inhibitors was much less pronounced in PC-9 cells, although phosphorylation levels of ERK1/2 in both cell lines were inhibited (Figure 5B). These results suggested that, in contrast to *EGFR* mutant lung cancer cells, both PI3K/Akt and MAPK signaling pathways are essential in lung cancer cells with wild-type *EGFR*.

## DISCUSSION

EGFR inhibitors have been recommended as the first choice for patients with advanced NSCLC that express *EGFR* activating mutations [29–31]. EGFR inhibitors can also prolong survival in patients with NSCLC after failure of chemotherapy regimens [20]. In the present study, we showed that gefitinib modestly inhibited cell viability of lung cancer cell lines, H358 and A549, through inhibition of EGFR phosphorylation and the downstream signaling molecules, Akt and ERK1/2. However, HGF reduced the sensitivity of these cells to gefitinib. Therefore, the influence of resistant mechanisms to EGFR-TKI sensitivity, such as high HGF levels, should be considered to improve patient prognosis.

*MET* amplification has been reported to induce gefitinib resistance via ErbB3 in *EGFR* mutant lung cancers [15]. However, we and other investigators have demonstrated that HGF induces gefitinib resistance mediated by MET, without the involvement of ErbB3 [13, 25]. Consistent with previous findings in *EGFR* mutant NSCLC, we found HGF suppressed sensitivity to gefitinib via the MET pathway without association to ErbB3 in wild-type *EGFR* NSCLC. Therefore, ligand-stimulated MET activation induces different downstream signals to gene amplification-stimulated MET activation in both NSCLCs harboring mutant and wild-type *EGFR*. The detailed mechanism of this difference needs to be clarified in future studies.

Studies have shown that most lung adenocarcinomas with wild-type *EGFR* were not highly sensitive to EGFR-TKIs [32–34]. However, even in lung cancer cells with wild-type *EGFR*, EGFR phosphorylation was inhibited by gefitinib, accompanied by modestly suppression of cell proliferation. Indeed, the first-generation of anti-EGFR therapies were all directed against the wild-type receptor [35]. Based on the findings of this study, lung cancers with wild-type *EGFR* are not addicted to EGFR signal, in contrast to *EGFR* mutant cells. Thus, at least in a group of lung cancers with wild-type *EGFR*, combining EGFR inhibition with the inhibition of other survival dependent signaling pathways might be a better therapeutic strategy, similar to the successful inhibition of lung cancers with *EGFR* mutations having acquired resistance [36].

The *KRA*S mutation has been used as a predictive marker of low sensitivity to EGFR-TKI in lung cancer cells harboring *EGFR* activating mutations [37]. The mechanism of *KRAS* mutation related resistance to EGFR-TKI has not been exclusively explained, though many investigators propose that mutant *KRAS* induces resistance to EGFR-TKI by activating MAPK pathways [38]. In the present study, analysis of A549 and H358 cells, which also express a *KRAS* mutant, further confirmed this concept. In the experiments of inhibition to downstream signaling pathways, PI3K inhibitors, but not MEK inhibitors, effectively suppressed proliferation of *EGFR* mutant PC-9 cells. In contrast, both PI3K inhibitors and MEK inhibitors showed inhibitory effects on the proliferation of A549 cells, indicating both downstream signaling pathways are involved in the proliferation of A549 cells.

In the present study, we found that HGF reduced the sensitivity to gefitinib, while the MET inhibitor, PHA-665752, restored the sensitivity. MET inhibitors have been researched in many clinical trials [39]. At present, crizotinib, a dual inhibitor of EML4-ALK and MET, already has been widely employed in lung cancer patients harboring the EML4-ALK fusion [40]. Based on our findings, crizotinib might be useful for this group of patients with lung cancers harboring wild-type *EGFR*. In summary, combined use of EGFR-TKI with a MET inhibitor or inhibition of downstream signaling molecules, such as PI3K or MEK, might be a better therapeutic alternative for at least a part of advanced lung cancer patients.

## MATERIALS AND METHODS

### Cell culture and reagents

The human lung adenocarcinoma cell lines H358 and A549 were purchased from Immuno-Biological Laboratories Co. and American Type Culture Collection, respectively. The human lung adenocarcinoma cell line PC-9 was purchased from Immuno-Biological Laboratories Co. (Gunma, Japan). H358, A549, and PC-9 cell lines were maintained in RPMI 1640 supplemented with 10% fetal bovine serum (FBS), 100 units/mL penicillin, and 100 units/mL streptomycin.

Gefitinib was obtained from AstraZeneca. Goat anti-human HGF neutralizing antibody and control goat IgG were purchased from R & D Systems. Erlotinib, PHA-665752, GSK2126458, PF04691502, AZD-8330 and TAK-733 were purchased from Selleck chemicals.

### Cell proliferation assay

Cell proliferation was measured using the 3-(4, 5-dimethylthiazol-2-yl)-2,5-diphenyl tetrazolium (MTT) dye reduction method. Tumor cells were plated at a density of 2 × 10^3^ cells/100 μL/well into 96-well plates in RPMI 1640 with 10% FBS. After 24-h incubation, various concentrations of gefitinib, PHA-665752, and/or HGF were added to each well, and incubation was continued for an additional 72 h. Then, 50 μL of MTT solution (2 mg/mL; Sigma) were added to all wells, and incubation was continued for another 2 h. The media containing MTT solution was removed, and the precipitated material was dissolved by adding 100 μL of DMSO. The absorbance was measured with a microplate reader at test and reference wavelengths of 490 and 570 nm, respectively. The percentage of growth is shown relative to untreated controls.

### Antibodies and western blotting

Western blotting was performed as previously described [41]. The primary antibodies used in this study were anti-Met (25H2), anti–phospho-Met (Y1234/Y1235) (3D7), anti–phospho EGFR (Y1068), anti-ErbB3 (1B2), anti–phospho-ErbB3 (Tyr1289) (21D3), anti-Akt, or phospho-Akt (Ser473) antibodies (1:1,000 dilution, Cell Signaling Technology), anti-human EGFR (1 μg/mL), anti-human/mouse/rat extracellular signal regulated kinase (ERK)-1/ERK2 (0.2 μg/mL), and anti–phospho ERK1/ERK2 (T202/Y204) (0.1 μg/mL) antibodies (R & D Systems).

### Assay for RNA interference and HGF gene transfection

Duplexed StealthRNAi (Invitrogen) targeted to MET and ErbB3 and Stealth RNAi Negative Control Low GC Duplex #3 (Invitrogen) were used for the RNA interference (RNAi) assay. The full-length HGF cDNA was cloned into an expression vector and used for the HGF gene transfection assay. One day before transfection, aliquots of 2 × 10^4^ tumor cells in 400 μL of antibiotic-free medium were plated into 24-well plates. After incubation for 24 h, the cells were transfected with small interfering RNA (siRNA; 50 μmol) or scramble RNA using Lipofectamine 2000 (1 μL) in accordance with the manufacturer's instructions. After 24 h incubation, the cells were washed with PBS and plated into 96-well plates. Cell proliferation in the presence or absence of gefitinib (I μmol/L) and/or recombinant human HGF (20 ng/nL) was measured with MTT as previously described. The sequences of siRNAs were as follows: MET, 5′-UCCAGAAGAUCAGUUUCCUAAUUCA-3′; ErbB3, 5′-GGCCAUGAAUGAAUUCUCUACUCUA-3′. Each experiment was done at least in triplicate in three independent experiments.

### HGF production

Cells (2 × 10^6^) were cultured in RPMI 1640 with 10% FBS for 24 h. The cells were washed with PBS and incubated for 48 h in RPMI 1640 with 10% FBS. Then, culture medium was harvested and centrifuged, and the supernatant was stored at −80°C until analysis. To determine the levels of HGF secreted, ELISA was done in accordance with the manufacturer's recommended procedures (Human HGF Quantikine ELISA. R & D systems). All samples were run in triplicate. Color intensity was measured at 450 nm. Growth factor concentrations were determined by comparison with standard curves. The detection limit was 40 pg/mL.

### Statistical analysis

All data are expressed as the mean ± SDs of experiments repeated at least three times. Significant differences between the means were measured by a two-tailed unpaired Student *t* test or one-way ANOVA. *P* < 0.05 was considered statistically significant.
